# Evaluation of Retinal Nerve Fiber Layer Thickness, Electroretinogram and Visual Evoked Potential in Patients with Alzheimer's Disease

**DOI:** 10.1155/2019/6248185

**Published:** 2019-12-26

**Authors:** Qi Zhe Ngoo, Wan Hazabbah Wan Hitam, Asrenee Ab Razak

**Affiliations:** ^1^Department of Ophthalmology, School of Medical Sciences, Health Campus, Universiti Sains Malaysia, 16150 Kubang Kerian, Kelantan, Malaysia; ^2^Department of Psychiatry, School of Medical Sciences, Health Campus, Universiti Sains Malaysia, 16150 Kota Bharu, Kelantan, Malaysia

## Abstract

**Objective:**

To study the retinal nerve fibre layer (RNFL) thickness and visual electrophysiology testing in patients with Alzheimer's disease (AD).

**Methods:**

A cross-sectional, hospital-based study: 25 AD subjects and 25 controls were recruited. Candidates who fulfil the criteria with normal ocular examinations were made to proceed with scanning laser polarimetry, pattern electroretinogram (PERG), and pattern visual evoked potential (PVEP) examinations of the right eye. RNFL thickness, PERG, and PVEP readings were evaluated.

**Results:**

In AD, the mean of average RNFL thickness was 45.28 *μ*m, SD = 3.61, *P* < 0.001 (*P* < 0.05), while the superior RNFL thickness was 54.44 *μ*m, SD = 2.85, *P* < 0.001 (*P* < 0.05) and inferior RNFL thickness was 47.11 *μ*m, SD = 4.52, *P* < 0.001 (*P* < 0.05). For PERG, the mean P50 latency was 63.88 ms, SD = 7.94, *P* < 0.001 (*P* < 0.05) and the mean amplitudes of P50 waves were 1.79 *μ*V, SD = 0.64, *P* < 0.001 (*P* < 0.05) and N95 waves were 2.43 *μ*V, SD = 0.90, *P* < 0.001 (*P* < 0.05). For PVEP, the mean latency of P100 was 119.00 ms, SD = 9.07, *P* < 0.001 (*P* < 0.05), while the mean latency of N135 was 145.20 ms, SD = 8.53, *P* < 0.001 (*P* < 0.05). The mean amplitude of P100 waves was 3.71 *μ*V, SD = 1.60, *P* < 0.001 (*P* < 0.05), whereas the mean amplitude of N135 waves was 3.67 *μ*V, SD = 2.02, *P* < 0.001 (*P* < 0.05). RNFL thickness strongly correlates with PERG readings, with P50 latency *R* = 0.582, *R*2 = 0.339, *P*=0.002 (*P* < 0.05), amplitude of P50 wave at *R* = 0.749, *R*2 = 0.561, *P* ≤ 0.001 (*P* < 0.05), and amplitude of N95 wave at *R* = 0.500, *R*2 = 0.250, *P*=0.011 (*P* < 0.05). No significant difference and correlation were observed on PVEP readings.

**Conclusion:**

The mean of the average, superior and inferior RNFL thickness were significantly lower in the AD group compared with control. There is also significant difference of PERG and PVEP parameters between AD and controls. Regression analysis showed average RNFL thickness having significantly linear relationship with the PERG parameters.

## 1. Introduction

Alzheimer's disease (AD) is a long-term progressive neurodegenerative disorder with large intersubject variability. The increase in the longevity of our population has contributed to the dramatic increase in neurodegenerative disorder. The prevalence of AD in Malaysia in 2015 was 1,23,000 people, and this number is expected to double by the year 2030 due to the increase in the number of people reaching old age [1].

Over the past few decades, various studies have found the link between AD and various visual disturbances. Patients with AD often complain of vision disturbances such as reading problem and blurring of vision. Patients might have normal visual acuity and normal fundus during routine ophthalmological examination [2, 3].

Loss of synapse between neurons in the cerebral cortex in AD may result in activation of the neurodegenerative pathway, which may contribute to the initiation and progression of cognitive impairment [4]. It is postulated that extracellular deposit of amyloid that forms plaque causes cerebral amyloid angiopathy and intraneuronal accumulations of Tau protein [5]. The plaque which is found in the brain of the patients with AD was also found in the eye [6]. This eventually causes cells apoptosis and loss of retinal cells, specifically the ganglion cells and theirs axons, ultimately causing optic nerve degeneration [7].

Optical coherence tomography (OCT) provides measurement of the thickness of the retinal nerve fibre layer (RNFL). RNFL thickness may reflect the morphological changes of the retina in AD [8]. It was found that RNFL thinning was observed in AD patients [9]. There are studies that demonstrate significant reductions in the total mean RNFL and also the superior and inferior quadrants of the RNFL in patients with mild and moderate AD [10].

Electrophysiological studies of the retina and optic nerve such as pattern electroretinography (PERG) and pattern visual evoked potential (PVEP) measure the bioelectrical activity of the retinal ganglion cells and optic nerve. The use of both PERG and PVEP at the same setting in AD has shown that visual impairment arises from the magnocellular pathway of visual processing [11]. Kamila Krasodomska et al. were able to demonstrate in a study that in patients with early stages of AD, statistically significant abnormalities were noted in PERG (implicit time increase in P50 wave, reduced amplitudes of P50 and N95 wave) and PVEP (Increase in latency of P100-wave) [12]. However till date, the available results are inconclusive as some cases reported normal PERG and PVEP test results.

Our study aimed to compare the difference between mean RNFL thickness, PVEP, and PERG in patients with mild to moderate AD and control. We also examined the potential relationship between RNFL thickness and PVEP and PERG. We were also taking into consideration other factors that can also contribute to RNFL thinning, age and gender which has not much been addressed in previous studies.

## 2. Materials and Methods

This observational cross-sectional study was conducted between June 2016 and February 2018. This study obtained ethical approval from the Human Research Ethics Committee, Universiti Sains Malaysia (USM/JEPeM/16010035) and was conducted in accordance with the Declaration of Helsinki for Human Research.

### 2.1. Patient Selection

Recruitment of AD patients was conducted in the Department of Psychiatry, Hospital Universiti Sains Malaysia. A total of 25 AD patients were recruited. Only newly diagnosed AD patients for whom treatment has not been commenced and met the criteria for diagnosis of AD, according to DSM-IV and DSM-V criteria after proper medical history review, physical examination, laboratory and neuroimaging evaluations, which include computed tomography scan (CT) of the brain to rule out vascular dementia, and neuropsychological testing, were selected. The Montreal Cognitive Assessment (MoCA) test was done to all recruited AD patients at the start of the study. Only AD patients with mild to moderate severity in accordance with MoCA score of 16 points and above are selected. The control group consists of 25 individuals presented to an ophthalmology clinic without systemic illnesses. All controls also underwent the MoCA test. Only AD patients and control subjects who have best corrected visual acuity of at least 6/12 using the Snellen chart and clear ocular media with refractive error of not more than ±4.0 dioptre were included in this study. Subjects who had a pre-existing optic neuropathy, retinopathy, maculopathy, history of trauma or previous ocular surgery, and systemic disease of neurological and demyelinating diseases were excluded. All participants who consented to take part in the study underwent thorough ocular examinations and fundus evaluation via slit lamp biomicroscopy (Topcon Corp, Japan). Intraocular pressure measurement was performed to rule out ocular pathology, which would have precluded participation in the study. All participants were then subjected for scanning laser polarimetry (SLP) examinations, PERG, and PVEP for the right eye.

### 2.2. Scanning Laser Polarimetry (SLP)

SLP examinations were performed using a GDx VCC SLP (Carl Zeiss Meditec, Inc., Dublin, CA, USA). The tests were performed by a single and well-trained operator. Only the test or repeated test that yielded signal strength of ≥6/10 was taken for interpretations to ensure accuracy of the results. Measurements were taken on the right eye which include average RNFL thickness, superior average of RNFL thickness, and inferior average of RNFL thickness.

### 2.3. Pattern ERG (PERG)

The PERG examinations were performd using a Granzfield PERG machine (Roland-Consult, RETI-port 32, Germany). The tests were performed by a single and well-trained operator. The PERG should be recorded without dilatation of pupils, with the ideal size of pupil not more than 3 mm, to preserve accommodation and quality of the retinal image. The patient needs to concentrate at a fixation mark in the centre of the screen at a node of the checkerboard. The checkerboard had two stimuli with large 8° (480 min of arc) and small 0.8° (48 min of arc) checks. The smaller checkerboard stimuli value was used in this study. Measurements were taken on the right eye which include peak latency values of N35, P50, and N95 and amplitude of N35-P50 and P50-N95. All the values and settings will be based on the International Society of Clinical Electrovisual Science (ISCEV) guideline standard.

### 2.4. Pattern VEP (PVEP)

The PVEP examinations were performed using a Granzfield PVEP machine (Roland-Consult, RETI-port 32, Germany). The tests were performed by a single and well-trained operator. PVEP checkerboard is elicited by checkerboard stimuli with large 1° (60 min of arc) and small 0.25° (15 min of arc) checks. It is the preferred procedure due to its relatively low variability of waveform and peak latency both within a subject and over a normal population. Measurements were taken on the right eye which include a positive peak latency component at approximately 100 ms (P100), proceeded by a negative peak latency (N75), and followed by a negative peak latency (N135). The amplitudes are N75- P100 and P100- N135. All the values and settings will be based on the ISCEV guideline standard.

### 2.5. Statistical Analysis

Data analysis was performed using the SPSS statistical package version 22 (SPSS, Chicago, IL, USA). Analysis was performed on the raw data collected. Descriptive analysis was used for the mean values and SD. All values were tested for normal distribution using the Shapiro–Wilk test in both groups. For demographic data, they will be tested for comparison of age, race, and gender. The Student's *t*-test and Pearson's chi-square test were used to analyze the demographic data. All *P* values of <0.05 were considered statistically significant. The independent *t*-test was used to compare the means of RNFL thickness, PERG, and PVEP implicit time and amplitudes between the study group and control. A *P* value of <0.05 was considered as significant. Pearson correlation was used to determine the correlations of RNFL thickness and the PERG and PVEP measurements. The strength of association was determined, and a *P* value of <0.05 was considered significant. General guidelines for assigning the strength of association by Cohen (1988) will be used (Table 1).

## 3. Results

Demographic data are shown in Table 2. There were a total of 50 participants. Among them 34 were male while 16 of them were female. The age of the participants ranged from 60 to 70 years. Forty-one of the participants were Malay, 6 were Chinese, and 3 were other races. The mean MoCA score for AD patients were 19.72, while control patients had higher mean score at 27.00. There were 13 AD patients with mild cognitive impairment and 12 patients with moderate cognitive impairment. All the control patients had normal cognitive function based on MoCA.

The mean RNFL values of AD patients and controls are shown in Table 3. We observed a significant difference in the mean of average RNFL thickness with *P* ≤ 0.001 and superior and inferior RNFL thickness with *P* ≤ 0.001 between the control and study group.

The MANCOVA analysis in Table 4 showed that age is a significant confounding factor affecting the RNFL thickness in our study. Overall retina nerve fibers analysis showed significant thinner in average, superior, and inferior RNFL thickness in the AD group compared with the control group.

Comparison of pattern ERG (PERG) readings between the group of AD and the control group is shown in Table 5. For the latency, there was significant difference (*P*=0.004) in P50 between the groups. However, for N35 and N95, there were no significant differences between the groups. For the amplitude of PERG, independent *t*-test showed significant differences for all amplitudes (*P* < 0.001).

The MANCOVA analysis (Table 6) showed that age is a confounder affecting the PERG readings in our study. Overall, the AD group showed significantly lower amplitude and prolonged latency of PERG readings compared to the control group.

Comparison of pattern VEP (PVEP) readings between the group of AD and the control group are shown in Table 7. For the latency, there was significant difference in P100 (*P* < 0.001) and N135 (*P* < 0.001) between the groups. However, for N75, there were no significant differences between the groups (*P*=0.284). For the amplitude of PVEP, the independent *t*-test showed significant differences for all amplitudes (*P* < 0.001).

The MANCOVA analysis (Table 8) showed that age is a confounder affecting the PVEP readings in our study. Overall, the AD group showed significantly lower amplitude and prolonged latency of PVEP readings compared with the control group.

Table 9 shows that there was a significantly strong negative correlation between RNFL thickness and PERG readings among the subject in the AD group in the P50 latency *R* = 0.582, *R*2 = 0.339, *r* = −0.582, *P*=0.030 (*P* < 0.05), strong positive correlation for amplitude N35–P50 *R* = 0.749, *R*2 = 0.561, *r* = 0.749, *P* < 0.001 (*P* < 0.05), and for amplitude P50–N95 *R* = 0.500, *R*2 = 0.250, *r* = 0.500, *P*=0.011 (*P* < 0.05). However, none of the PVEP parameters had significant linear relationship with the RNFL thickness among subjects in the AD group.

## 4. Discussion

Alzheimer's disease is a chronic and progressive neurodegenerative disease which involves both genetic and environmental factors [13]. The disease process causes the loss of synapse between neurons in the cerebral cortex which leads to cognitive impairment [4]. The loss of synapse is largely caused by extracellular deposit of amyloid that forms plaque which later causes cerebral amyloid angiopathy and intraneuronal accumulations of neurofibrillary tangles known as Tau protein [5]. In ocular tissues, the same pathophysiology occurs, causing optic nerve degeneration with loss of retinal cells, specifically the ganglion cells and theirs axons which are profound [7]. This retrograde damage of optic nerve neurons may serve as an early marker for neurodegeneration in AD as it can occur prior to hippocampal damage that causes cognitive dysfunction [14].

Our result showed a significant difference in the average RNFL thickness with a mean difference of 9.100, *P* < 0.001 (*P* < 0.05), superior thickness with a mean difference of 5.920, *P* < 0.001 (*P* < 0.05), and inferior thickness with a mean difference of 5.528, *P* < 0.001 (*P* < 0.05) between the control and study group. This is similar to the results of Kergoat et al. [15] who found significant thinning of RNFL in all quadrants in AD subjects and Liu et al. [10] who found significant thinning in the superior and inferior quadrants. This may be due to the fact that there are higher numbers of neurons in superior and inferior quadrants which then causes more prominent neurodegeneration. Likewise, similar findings were also reported by Parisi et al. [9]. Bruban demonstrated that beta amyloid deposit in the retinal cells may lead to RNFL thinning [6]. Reduction of acetylcholine in AD disrupts the normal function of retinal cells causing loss of ganglion cells and axons which eventually leads to optic nerve degeneration [16].

We went a step further by performing a multivariate analysis of covariant (MANCOVA) to determine whether our significant result was also contributed by any confounding factor. It has been shown in a previous study that RNFL thickness was reduced as age increased [17]. In our study, age was found to be a significant confounder.

We found a significant difference with regards to PERG parameters between AD and control. The P50 latency showed significant difference with a mean difference of −9.12, *P* < 0.001 (*P* < 0.05), whereas the amplitude of P50 wave had a mean difference of 2.7, *P* < 0.001 (*P* < 0.05) and amplitude of N95 wave had a mean difference of 3.78, *P* < 0.001 (*P* < 0.05). This is similar to the study by Katz et al. who found significant differences of PERG parameters between AD and controls [18]. Similar results were obtained by Kamila et al. in which the latency of P50 wave was increased and the amplitude of P50 and N95 waves was significantly reduced [12]. This is most likely due to the disturbance of the normal functional properties of the ganglion cells which are largely represented by P50 and N95 waves [19]. The prolonged latency time of P50 is also associated with not only the dysfunction of ganglion cells but also the outer layers of retina which are closely related to ganglion cells [20]. However, there were contradictory findings in the study conducted by Iseri et al. in which they found no statistically difference for PERG results between patients with AD and controls [21]. They postulated that this probably happen due to small sample size as it was difficult to recruit the patients who are co-operative and have minimal cognitive dysfunctions. As such, we limit our samples to include only patients with mild to moderate AD in order to overcome this limitation. We have almost half of our sample in the moderate AD group, who are all very co-operative to complete the test.

In the present study, we found that PERG response is most likely affected by age. Acceleration of degenerative process rate of retina ganglion cells is more pronounced in 60 years of age and above [22]. However, since our recruited patients were age matched to the controls, there was no significant difference in age distribution of AD and controls. MANCOVA analysis showed that age was a significant confounder. However, there was still significant difference of PERG response between AD and controls. Thus, there is high possibility that the changes in PERG are due to AD.

There was decrease in magnitude of the amplitude and increase in latency of P100 and N135 of PVEP in AD patients in the present study. The P100 latency showed significant difference with a mean difference of −12.32, *P* < 0.001 (*P* < 0.05) and N135 latency with mean difference of −15.00, *P* < 0.001 (*P* < 0.05). The amplitude of P100 wave had a mean difference of 6.07, *P* < 0.001 (*P* < 0.05), and amplitude of N135 wave had a mean difference of 6.85, *P* < 0.001 (*P* < 0.05). Pollock et al. reported increase in latency of P100 and N135 in AD patients [23]. Similar observations were also seen in a study done by Partanen et al. [24]. The delay in the latency of P100 wave can be a subclinical feature of optic nerve dysfunction [25]. This is because P100 wave represents the bioelectrical function of the optic nerve [26]. In another study conducted by Kromer et al., there was significant reduction of amplitude of P100 and N135 waves [27]. This is contrary to the study done by Kamila et al., in which there was no statistically significant difference in the amplitude of P100 and N135 waves between AD patients and control [12]. This was largely due to small sample size, and most of their patients were in the early stages of AD in which there is lack of regional differentiation in PVEP response between foveal and parafoveal part of the retina. Our study showed decrease in amplitude of P100 and N135 wave which was in line with the postulated pathogenesis of the disease that involves the dysfunction in the magnocellular pathway which affects the neural transmission [28, 29].

For PVEP measurements, we went a step further by performing a MANCOVA analysis to determine whether our significant result was also contributed by any confounding factor. It has been shown in a previous study that PVEP amplitudes will reduce and latency will increase as age increased [30]. This may be due to loss of neurons and changes in the production of neurotransmitter as the age increased. In our study, age was found to be a significant confounder.

Our regression analysis showed a strongly significant negative direct linear relationship between the P50 latency in PERG with the average RNFL thickness. For every decrement of 1 *μ*m of RNFL thickness, there is a delay of 0.582 ms occurred in the P50 implicit time. There is also a strongly significant positive direct linear relationship between the amplitudes of P50 waves and N95 waves with the average RNFL thickness. For every decrement of 1 *μ*m of RNFL thickness, there is a decrease in the amplitude of 0.749 *μ*V of P50 wave and 0.500 *μ*V decrease in the N95 wave. These results are similar to studies by Parisi et al. and Doustar et al. [31, 32] who also found correlation between the PERG parameters, particularly the P50 latency, P50, and N95 waves amplitude with average RNFL thickness. This is most likely due to the reduction in the RNFL thickness causing hindrance in the visual performance which is measured by PERG [33]. Reduction in the “b” wave or P50 wave particularly correlates to the reduction of ganglion cells in the retina of patients with AD [34]. However, in our study, there are no significant correlations and associations between any of the PVEP parameters and average RNFL thickness. This is in line with the study conducted by Iseri et al. in which there was no significant correlation in readings of PVEP with RNFL thickness [21].

In our study, we concluded that abnormalities in RNFL parameters occur secondary to RNFL loss; thus, RNFL changes can be a sensitive biomarker of neurodegeneration in AD. As there is overlapping of RNFL abnormalities found in other neurodegenerative diseases, other investigations of AD effect on certain parts of the retina remain crucial to be performed to enhance the practicality of RNFL parameters in diagnosis of AD. Thus, the use of PERG and PVEP in our study provides more depth regarding optic nerve dysfunction due to the loss of neurons.

We are unable to determine the exact duration of the AD. However, majority of our subjects were diagnosed after having cognitive impairment within 2 years of diagnosis when we performed examination on them. It is possible that the greater the severity and longer duration of the AD, the greater the RNFL and electrophysiological alterations [35].

Our study is a cross-sectional study. There were no comparisons conducted or follow-up to actually look at the changes in all of the studied parameters over time. We suggest that in future, a prospective study to be conducted on these patients in order to evaluate the possible changes on the RNFL thickness as well as PERG and PVEP parameters in these patients.

Our study included patients who are newly diagnosed. This is important as we excluded the possible effect of AD medications such as the cholinesterase inhibitor group. These drugs might aid in stabilizing the progression of the disease process, even though the possible benefits towards RNFL and visual pathway are still lack in evidence [36].

Our study is limited by the presence of cognitive dysfunction in our AD patients who some are not co-operative to undergo examinations. We were able to fully exclude those with severe cognitive dysfunction. Therefore, only patients with mild to moderate AD are included in the study. We suggest that if similar study is to be carried out in the future, the patients in the severe group should be selected and the use of more patient friendly device might be advocated to aid in their examinations.

## 5. Conclusions

There were a few limitations in our study. Firstly, being unable to correlate the neurophysiological test with the neurocognitive profiles which comprise of the severity of the AD due to time constraint and the limited number of patients. As we were unable to recruit the patients with the severe form of AD, the correlation between the cognitive profiles and the physiological changes could not be performed. Secondly, the electrophysiological test only used the stimuli of black and white checker box for testing in PVEP and PERG due to limitation of machine in our setting. The use of different visual stimuli such as chromatic or black-white sinusoidal pattern can demonstrate the pathology in different visual pathway, namely the magnocellular and parvocellular pathway of the brain. Future studies should use upgraded machine to look into parameters generated in these pathways.

Our study showed that AD patients have reduced retinal nerve fibre layer thickness in the average, superior and inferior quadrant compared with controls. There is also significant difference of PERG and PVEP parameters between AD and controls. AD patients have a reduction of PERG amplitudes and prolonged latency of P50 waves. There is also significant reduction of amplitude of PVEP and prolonged latency of P100 and N135 waves. A significant strong direct negative correlation exists between P50 wave latency as well as a strong direct positive correlation in the amplitude of P50 and N95 waves with RNFL thickness. Further evaluations and long-term follow-up may be needed in future in order to monitor the progression of RNFL thinning and alteration of electrophysiological studies in this group of patients.

## Figures and Tables

**Table 1:** Cohen strength of association.

Coefficient value	Strength of association
0.1 < *r* < 0.3	Small/weak correlation
0.3 < *r* < 0.5	Fair/moderate correlation
*R* > 0.5	Large/strong correlation

**Table 2:** Demographic data of Alzheimer's disease (AD) subjects and control.

	AD *N* = 25	Control *N* = 25	*P* value
Mean age (mean, SD)	65.52 (2.79)	64.28 (3.42)	0.166^a^

Sex (*n*, %)			
Male	13 (52.0)	21 (84.0)	0.015^b^
Female	12 (48.0)	4 (16.0)	

Race (*n*, %)			
Malay	19 (76.0)	22 (88.0)	0.543^b^
Chinese	4 (4.0)	2 (8.0)	
Others	2 (8.0)	1 (4.0)	

Mean MoCA score (mean, SD)	19.72 (3.64)	27.00 (1.61)	<0.001^a^

MoCA score classification (*n*, %)			
Normal	0 (0)	25 (100)	<0.001^b^
Mild cognitive impairment	13 (52.0)	0 (0)	
Mod cognitive impairment	12 (48.0)	0 (0)	

^a^Independent *t*-test. ^b^Pearson's chi-square test.

**Table 3:** Comparison of mean retinal nerve fibre layer thickness of the right eye between AD patients and control.

RNFL thickness (*μ*m)	AD (*N* = 25) mean (SD)	Control (*N* = 25) mean (SD)	*t*	Means difference	Confidence interval 95%		df	*P* value^*∗*^
					Lower	Upper		
Average	45.28 (3.61)	54.38 (5.20)	7.190	9.100	6.547	11.653	42.80	<0.001
Superior	54.44 (2.85)	60.36 (4.11)	5.925	5.920	3.905	7.935	42.73	<0.001
Inferior	47.11 (4.52)	52.64 (4.27)	4.444	5.528	3.027	8.028	48.00	<0.001

^*∗*^Independent *t*-test.

**Table 4:** Multivariate analysis of covariant (MANCOVA) of the right eye RNFL thickness in AD subject and control.

Effect	Value	*F*	Hypothesis df	Error df	Partial etan square	Significance
Age	0.471	16.864	3.00	45.00	0.529	<0.001

**Table 5:** Comparison of PERG readings of the right eye between AD patients and control.

PERG parameters	AD (*N* = 25) mean (SD)	Control (*N* = 25) mean (SD)	*t*	Means difference	Confidence interval 95%		df	*P* value^*∗*^
					Lower	Upper		
N35 (ms)	40.32 (6.63)	36.44 (8.03)	−1.863	−3.88	−8.067	0.307	48.00	0.069
P50 (ms)	63.88 (7.94)	54.76 (8.24)	−3.985	−9.12	−13.722	−4.518	48.00	<0.001
N95 (ms)	107.96 (8.01)	106.64 (8.59)	−0.562	−1.32	−6.044	3.404	48.00	0.577
P50 wave (*μ*V)	1.79 (0.64)	4.49 (1.44)	8.574	2.70	2.062	3.345	33.17	<0.001
N95 wave (*μ*V)	2.43 (0.90)	6.21 (2.65)	6.756	3.78	2.634	4.919	29.43	<0.001

^*∗*^Independent *t*-test.

**Table 6:** Multivariate analysis of covariant (MANCOVA) of the right eye PERG readings in AD subject and control.

Effect	Value	*F*	Hypothesis df	Error df	Partial etan square	Significance
Age	0.359	15.364	5.00	43.00	0.641	<0.001

**Table 7:** Comparison of PVEP reading of the right eye between AD patients and control.

PVEP parameters	AD (*N* = 25) mean (SD)	Control (*N* = 25) mean (SD)	*t*	Means difference	Confidence interval 95%		df	*P* value^*∗*^
					Lower	Upper		
N75 (ms)	77.40 (11.03)	74.04 (10.88)	−1.085	−3.36	−9.589	2.869	48.00	0.284
P100 (ms)	119.00 (9.07)	106.68 (10.53)	−4.431	−12.32	−17.910	−6.730	48.00	<0.001
N135 (ms)	145.20 (8.53)	130.20 (7.94)	−6.437	−15.00	−19.685	−10.315	48.00	<0.001
P100 -wave (*μ*V)	3.71 (1.60)	9.78 (2.82)	9.363	6.07	4.760	7.386	37.94	<0.001
N135 -wave (*μ*V)	3.67 (2.02)	10.52 (3.49)	8.498	6.85	5.218	8.481	38.52	<0.001

^*∗*^Independent *t*-test.

**Table 8:** Multivariate analysis of covariant (MANCOVA) of the right eye PVEP readings in AD subject and control.

Effect	Value	*F*	Hypothesis df	Error df	Partial etan square	Significance
Age	0.208	32.763	5.00	43.00	0.792	<0.001

**Table 9:** Correlation between the mean RNFL thickness of the right eye with PERG and PVEP readings in AD patients.

	*R*	*R* ^2^	*F*	*r*	*P* value^*∗*^
PERG parameters					
N35 (ms)	0.013	<0.001	0.004	0.013	0.951
P50 (ms)	0.582	0.339	11.795	−0.582	0.002^*∗*^
N95 (ms)	0.114	0.013	0.303	0.114	0.587
N35–P50 (*μ*V)	0.749	0.561	29.388	0.749	<0.001^*∗*^
P50–N95 (*μ*V)	0.500	0.250	7.667	0.500	0.011^*∗*^

PVEP parameters					
N75 (ms)	0.036	0.001	0.030	−0.036	0.865
P100 (ms)	0.282	0.079	1.986	0.282	0.172
N135 (ms)	0.301	0.091	2.300	−0.301	0.143
N75–P100 (*μ*V)	0.024	0.001	0.013	−0.024	0.910
P100–N135 (*μ*V)	0.316	0.100	2.555	−0.316	0.124

Simple linear regression and Pearson's correlation (*P* < 0.05).

## Data Availability

The SPSS data used to support the findings of this study are available from the corresponding author upon request.
